# Against the stream: intermittent nurse observations of in-patients at night serve no purpose and cause sleep deprivation

**DOI:** 10.1192/bjb.2018.116

**Published:** 2019-08

**Authors:** David Veale

**Affiliations:** 1 South London and Maudsley NHS Trust; 2 Institute of Psychiatry, Psychology and Neuroscience

**Keywords:** In-patient treatment, suicide, psychiatric nursing, risk assessment

## Abstract

This paper argues that intermittent nursing observations of in-patients at night do not reduce the risk of suicide or severe self-harm. Suicides between 23.00 h and 07.00 h are rare, and these overwhelmingly occur under intermittent observations. Such observation is purely a defensive intervention to document that a patient is safe at a particular time, as there is no engagement. For the large majority of in-patients, it has the unintended consequence of causing sleep deprivation. The intervention may cause harm to in-patients by making their disorder worse and increase their risk during the day. If patients are judged to be at immediate risk, then they should be placed on constant observation. If they are not, then optimising sleep is important for treating a psychiatric disorder and they should be placed on general observations.

**Declaration of interest:**

None.

Nursing observation is defined as ‘… regarding the patient attentively, minimizing the extent to which they feel they are under surveillance, encouraging communication, listening, and conveying to the patient that they are valued and cared for …’.^1^ National Institute for Health and Care Excellence guidelines^2^ define various levels of observation determined by a risk assessment, especially for severe self-harm, suicide, violence and absconding. These include *continuous* observation by one or more staff; low-level *intermittent* observation (usually a frequency of once or twice an hour); and high-level *intermittent* observation (usually two to four times an hour). The term *general* observations usually refers to a frequency of once or twice in a staff shift. The importance of ‘engagement’ (that is, emotional and psychological containment of distress and giving of hope) is emphasised as a genuine (not just linguistic alternative) to observation.^3^ National guidance and local policies assume that nursing observation operates over 24 h. As I shall explain, this is where the problem lies: ‘engagement’ is not required at night when a patient needs to sleep and to be kept safe.

It is up to the nursing staff to determine what fulfils ‘reasonable’ observation when patients are sleeping at night. A variety of practices and frequency of observations are used at night to document that a patient is safe at a certain time. Policies usually require the staff member to clearly see the patient is breathing. At night this typically involves either opening the window hatch in the door or entering the bedroom and shining a torch on the patient's face or switching on a light or waking the patient by shaking them to see that they are still breathing. There are many complaints by patients on the practice as it disturbs their sleep and they are frequently unable to get back to sleep. However, the documentation of a patient's safety has become the only metric of importance to regulators and managers. No research or discussion has ever been published into the effectiveness for risk management of nursing observations at night. However, I will argue that the process of documentation by intermittent observations at night has many unintended consequences.

Surveys of psychiatric in-patients find that the large majority experience insomnia because of the noise and light on the ward and from nursing observations.^4^^,^^5^ There is a bidirectional relationship so that insomnia is not just a symptom of psychiatric disorder – intermittent sleep deprivation makes most psychiatric disorders worse.^6^ Importantly, sleep duration is negatively correlated with subsequent length of time in hospital^7^ and is associated with a range of physical and mental health problems.^8^ Specifically, sleep deprivation leads to negative changes in the neuroendocrine, immune and inflammatory systems, as well as hypertension. Furthermore, evidence from correlational and experimental studies have demonstrated that reduced sleep has a severe effect on emotional regulation.^9^ Paradoxically, it would be better to have one night of continuous sleep deprivation, followed by advancing the time of sleep over 3 days combined with bright light in the mornings (called ‘triple chronotherapy’). This resets the circadian rhythm and can lead to a rapid improvement in risk of suicide and improvement in mood.^10^^,^^11^

Sleep deprivation and worsening of symptoms for the many might be justified if it significantly reduced the frequency of suicide or severe self-harm. However, the effectiveness of intermittent observations at night in preventing suicide is highly questionable. The most recent National Confidential Enquiry into Suicide and Homicide^12^ reported that there were 114 suicides by in-patients in the previous year (compared with 1600 suicides *per annum* by people known to psychiatric services and about 6200 people *per annum* in the community who are not known). About one-third of the in-patient deaths occurred during busy periods on the ward (e.g. 07.00–09.00 h, 13.00–15.00 h and 19.00–21.00 h), hours during handover or when staff had multiple duties to attend to.^12^ I requested a further search from the National Confidential Inquiry on the numbers of in-patients who died between 23.00 h and 07.00 h. Between 2011–2016, there were 464 in-patient suicides. Of these, 54 out of 338 (16%, excluding unknowns) died at night (about half on the ward, about half off the ward). There are no statistics collected on the frequency of severe self-harm. However, it would be surprising if the pattern of self-harm were very different to the reduced risk of suicide between 23.00 h and 07.00 h.

What is also known is that 91% of in-patient deaths by suicide occur under *intermittent* rather than constant observation.^13^ This is not really surprising. If you were determined to end your life or self-harm while being observed intermittently, you would choose a time just after you have been observed and then act. The rate of suicide is 13.7 per 10 000 admissions (0.14%).^14^ Therefore, about two out of 10 000 admissions die at night between 23.00 h and 07.00 h, and these are overwhelmingly under *intermittent* observation. It is true that because there are no randomised controlled trials (RCTs) we do not know how many deaths were prevented (or delayed) by intermittent observations. Owing to of the very low rate of suicide at night (two per 10 000 admissions), there will never be a RCT of, say, intermittent *v.* general observations at night with mortality as the primary outcome, because the numbers required would be about 250 000 to demonstrate non-inferiority between two groups.

My conclusion is that intermittent observations at night currently cause sleep deprivation for the majority of in-patients. They appear to do little to prevent suicide and are just as likely to be increasing risk during the day and prolonging in-patient stays. The best way of managing risk is to treat the disorder, and this includes insomnia. When I discuss this with colleagues, I find a widespread view that the practice stems from institutions’ fear that something may go wrong, and that the staff or hospital may be criticised by a coroner or regulator. When a serious event occurs, the monetary as well as the human costs can be large, and thus the best defence against possible negligence claims is for staff to follow local policies or national guidelines. However, guidelines that assume the same frequency of intermittent observations during the day or night do not make sense because (a) staff are not expected to engage a patient at night; (b) suicide is much less common at night; and (c) when suicide does occur, it is generally under intermittent observations. Night-time observations thus cannot be justified on the grounds of keeping patients safe and may increase risk over the following days by causing sleep deprivation.^15^

If a patient is assessed as an immediate risk to themselves or others, then they should of course be put under *constant* observation at night – I do not have a problem with this, with the caveat that deciding who is at ‘immediate risk’ is unreliable at predicting suicide. There is no evidence for differentiating between low, medium and high risk in psychiatric in-patients.^16^ In brief, there is an overwhelming number of false positives from those rated as having a high risk and just as many suicides from those rated as a low or medium risk. This is important because such ratings determine the level of observations during the day *and* at night. Balancing reasonableness and proportionality are crucial issues in observations at night. Thus, a policy of placing a patient under intermittent observations at night because they are rated as low or medium risk allows an institution to feel they are doing something to manage risk and protect themselves from criticism from regulators or negligence claims.

I would like to see several areas of change. First, observations should be routinely called ‘engagement’ during the day and ‘observations’ by night. Second, national guidelines on engagement and observation should take into account that only a minority of suicides occur at night. Then it should be possible to have a personalised care plan so that the frequency of engagement during the day and observations at night can be varied if the risk is assessed as differing according to context. Thus, patients who are on intermittent once-an-hour engagement during the day may be put on general observations at night. This should not stop staff being inquisitive and vigilant when they believe something is ‘not right’.^1^ Last, I would like sleep on an in-patient ward to be taken seriously, and the principles of sleep hygiene and cognitive–behavioural therapy for insomnia, adapted for a psychiatric ward, to be implemented.^17^ This means focusing the culture and environment of a ward on optimising sleep at night and therefore managing risk by treating the disorder through improving sleep. We need to listen to patients and develop innovative solutions to improve care at night. Finally, duration of stay, global severity of psychiatric disorder and quality of sleep should be important indicators for RCTs, single case experimental designs and quality improvement projects on observations.

## About the author

**David Veale** is a Consultant Psychiatrist at the Centre for Anxiety Disorders and Trauma at the South London and Maudsley NHS Foundation Trust and Visiting Professor in Cognitive Behavioural Psychotherapies at the Institute of Psychiatry, Psychology and Neuroscience, Kings College London, UK.
